# Birth as a neuro-psycho-social event: An integrative model of maternal experiences and their relation to neurohormonal events during childbirth

**DOI:** 10.1371/journal.pone.0230992

**Published:** 2020-07-28

**Authors:** Ibone Olza, Kerstin Uvnas-Moberg, Anette Ekström-Bergström, Patricia Leahy-Warren, Sigfridur Inga Karlsdottir, Marianne Nieuwenhuijze, Stella Villarmea, Eleni Hadjigeorgiou, Maria Kazmierczak, Andria Spyridou, Sarah Buckley

**Affiliations:** 1 Faculty of Medicine, University of Alcalá, Henares, Spain; 2 Swedish University of Agricultural Sciences, Skara, Sweden; 3 Department of Health Sciences, University of West, Trollhättan, Sweden; 4 School of Nursing and Midwifery, University College, Cork, Ireland; 5 School of Health Sciences, University of Akureyri, Akureyri, Iceland; 6 Research Centre for Midwifery Science Maastricht, Zuyd University, Heerlen, The Netherlands; 7 Faculty of Philosophy, University of Alcalá, Henares, Spain; 8 Faculty of Philosophy, University of Oxford, Oxford, United Kingdom; 9 Nursing Department, Faculty of Health Sciences, Cyprus University of Technology, Limassol, Cyprus; 10 Institute of Psychology, Uniwersytet Gdanski, Gdansk, Poland; 11 Nursing Department, Faculty of Health Sciences, Cyprus University of Technology, Limassol, Cyprus; 12 School of Public Health, The University of Queensland, Herston, Qld, Australia; World Health Organization, SWITZERLAND

## Abstract

**Background:**

Psychological aspects of labor and birth have received little attention within maternity care service planning or clinical practice. The aim of this paper is to propose a model demonstrating how neurohormonal processes, in particular oxytocinergic mechanisms, not only control the physiological aspects of labor and birth, but also contribute to the subjective psychological experiences of birth. In addition, sensory information from the uterus as well as the external environment might influence these neurohormonal processes thereby influencing the progress of labor and the experience of birth.

**Methodology:**

In this new model of childbirth, we integrated the findings from two previous systematic reviews, one on maternal plasma levels of oxytocin during physiological childbirth and one meta-synthesis of women´s subjective experiences of physiological childbirth.

**Findings:**

The neurobiological processes induced by the release of endogenous oxytocin during birth influence maternal behaviour and feelings in connection with birth in order to facilitate birth. The psychological experiences during birth may promote an optimal transition to motherhood. The spontaneous altered state of consciousness, that some women experience, may well be a hallmark of physiological childbirth in humans. The data also highlights the crucial role of one-to-one support during labor and birth. The physiological importance of social support to reduce labor stress and pain necessitates a reconsideration of many aspects of modern maternity care.

**Conclusion:**

By listening to women’s experiences and by observing women during childbirth, factors that contribute to an optimized process of labor, such as the mothers’ wellbeing and feelings of safety, may be identified. These observations support the integrative role of endogenous oxytocin in coordinating the neuroendocrine, psychological and physiological aspects of labor and birth, including oxytocin mediated. decrease of pain, fear and stress, support the need for midwifery one-to-one support in labour as well as the need for maternity care that optimizes the function of these neuroendocrine processes even when birth interventions are used. Women and their partners would benefit from understanding the crucial role that endogenous oxytocin plays in the psychological and neuroendocrinological process of labor.

## Introduction

Women’s experiences of maternity care are a public health issue worldwide as acknowledged by the World Health Organization (WHO) in their 2018 report, *Intrapartum Care for a Positive Childbirth Experience* [1]. In some places women and babies still die because of lack of professional care, while in others they suffer from ineffective or harmful unnecessary interventions related to the medicalization of childbirth [2]. The WHO’s recent recommendations establish women´s experience of care as a critical aspect of high-quality maternity care, and not just a complement to the provision of clinical practices. This report defines a positive childbirth experience as “*… one that fulfills or exceeds a woman´s prior personal and sociocultural beliefs and expectations and includes giving birth to a healthy baby in a clinically and psychologically safe environment with continuity of care and emotional support*” [1].

Understanding what constitutes a psychologically safe environment to give birth requires listening to and learning from women´s experiences of childbirth [3]. Psychological aspects of labor and birth have generally received little attention in maternity service care planning or clinical practice. The processes of labor and birth are still largely viewed as the physiological process by which labor progress and are evaluated by external measures, such as the level of cervical dilation, which requires women to undergo repeated vaginal examinations [4]. In addition, the WHO partograph relies on measures as the main assessment of progress of labor. The mechanistic model of birth is associated with a medicalized and ‘technocratic’ approach to maternity care [5]. However, birth is more than a mechanical process by which the baby is transferred from the uterus to the outside world. It also comprises the physiological and psychological adaptations that facilitate and optimize birth outcome for mother and baby and, also promote long-term health and wellbeing for both by stimulating interaction and bonding.

From this wider perspective, birth can be understood as a neuroendocrinological event, orchestrated by neurohormones produced in both the mother and fetus and which influence the function of the brain and the body [6]. From prodromal labor to the early postpartum period, both mother and baby are exposed to a very specifically organized neurochemical cascade. Neurohormonal processes influence the progress of labor, including the mother’s psychological experiences of labor and birth. This cascade facilitates the reduction of pain and stress levels during and after birth and stimulates the interaction and bonding between mother and baby in the postpartum period [7].

Social and cultural perspectives on childbirth are also illuminating [8]. These include a wide variety of perspectives and critiques. For example, the feminist critique describes the organization of childbirth services and care as a gendered and patriarchal process that reinforces female inferiority [9,10]. Others have conceptualized childbirth as an institutional phenomenon influenced by power relations and structural dynamics [8]. Many approaches emphasize that an alternative theoretical framework is needed in order to promote humanization in childbirth practices [11]. Midwives are recognized globally as the most appropriate maternity care-giver for healthy mothers and babies [12]. Continuity of midwifery care offers better outcomes compared to other models of care, including fewer preterm births, less use of interventions and greater maternal satisfaction [13]. In some countries, labor support is provided by other types of maternity caregivers, including nurses and obstetricians. Midwives and many maternity care providers support humanistic care, which does not exclude the use of medical interventions when required [14].

In recent years, researchers have started to study women´s experiences of physiological births and attempts have been made to relate women´s behaviors and emotions during childbirth to neurohormonal processes in particular the stress system [15]. A more detailed understanding of the neurohormonal mechanisms including the role of effects of oxytocin in the brain, and the parallel effects on women’s experiences of labor and birth, will help care providers to fulfill women’s needs for a psychologically safe and positive experience [16].

Thus, there is a need for a new model of childbirth care that integrates neuroendocrinological, physiological and psychosocial understanding of labor and birth and that is based on a salutogenic and health promotion perspectives [17]. Such an approach would promote a healthy and satisfying experience of childbirth, not only for women, babies and their families but also for maternity care providers.

## Aim of the paper

In this overview, we propose a new model of childbirth that integrates neuroendocrinological, physiological and psychosocial processes during labor including the subjective experiences of women who have had a physiological childbirth.

## Methodology

Two independent systematic reviews were concurrently undertaken (as a part of the work within European Union funded COST Action IS 1405 entitled B.I.R.T.H: Building Intrapartum Research Through Health). The first paper reviewed maternal plasma levels of oxytocin during physiological childbirth and also presented the association between plasma levels of oxytocin and actions of oxytocin mediated by nervous mechanisms in the brain during labor and birth, which we summarize in section 1 [18]. The second paper is a meta-synthesis of women´s own reports on their lived experiences of physiological childbirth, which we summarize in section 2 [19]. Both publications were peer reviewed and published.

In the present third paper we apply the data obtained from the two first papers in order to develop an integrative model including neuroendocrinology, physiology and psychology. We will first briefly summarize the findings of the two previous papers and then, in section 3, propose a model for how neurohormonal processes, in particular oxytocinergic mechanisms, are involved in the psychological experiences of childbirth and also the mothers’ behavior and physiology. Special attention is given to the clinical implications of our analysis for healthcare professionals providing physical, psychological and mental care to women during and after childbirth. Finally, we propose suggestions for further research and implementation of this knowledge.

## Findings

In brief the information in paragraph 1 is cited from a paper by Uvnäs Moberg et al., 2019 [18], and the material in paragraph 2 is taken from data reported in paper by Olza et al., 2018 [19]. Full references are given within the original papers [18,19].

### 1.1 The release of endogenous oxytocin during physiological childbirth (data cited and main references Uvnäs Moberg et al., 2019 [18])

During pregnancy the maternal body and brain undergoes a profound and lasting transformation in order to facilitate birth and motherhood [20]. Oxytocin is produced in the magnocellular neurons of the Supraoptic (SON) and the Paraventricular (PVN) nuclei of the hypothalamus and transported to the posterior pituitary from where it is released into the circulation. Oxytocin released within the brain influences neuroendocrinological, physiological and psychological processes during labor, birth, and the early post-partum period. Levels of oxytocin and number of oxytocin receptors, increase in the uterus during pregnancy in response to the increasing levels of estrogen [21,22]. The oxytocin system is highly activated by the end of pregnancy. As also summarized in the previous paper by Uvnäs Moberg et al., 2019, (all references are given in this paper) the levels of oxytocin rise during labor, which stimulates uterine contractions and contributes to the opening of the birth canal. As labor proceeds, oxytocin pulses increase in frequency, amplitude and duration, and at the moment of birth, oxytocin levels are 3–4 times higher than in the beginning of the labor. As also summarized in the previous paper by Uvnäs Moberg et al., 2019, and all references are given in this paper [18], oxytocin is additionally released into the brain during birth from dendrites and cell bodies of the magnocellular neurons within the SON and PVN, as well as from short nerve branches (axon collaterals of these neurons), and from oxytocin containing nerves originating from parvocellular neurons in the PVN) and which innervate important regulatory areas in the brain [18].

### 1.2 Interaction between the oxytocin and the stress systems during labor (data cited and main references from Uvnäs Moberg et al., 2019 [18])

The uterus is innervated by the autonomic nervous system and efferent or outgoing parasympathetic and sympathetic nerve fibres influence the function of the uterus. In addition, afferent or ingoing sensory fibres send information to the brain regarding the state of the uterus. Oxytocin released within the brain during labor and birth induces pain relief, decreases fear and stress levels and stimulates social interactive behaviors. Oxytocin fibers that project from the brain down to parasympathetic networks (plexa) in the lumbo-sacral region of the spinal cord are also activated and contribute to stimulation of uterine contractions and of blood flow to the uterus. Activation of the ingoing parasympathetic fibres from the uterus to the brain increase oxytocin release. These nerves are activated, when the fetus’s head presses on the cervix and the vagina (known as the Ferguson reflex) and results in an increased release of oxytocin from the SON and PVN of the hypothalamus. As the circulating levels of oxytocin increase, the frequency of uterine contractions increases and consequently the pressure exerted by the fetus´ head increases. In this way a feed forward process is initiated. When oxytocin is released into the brain as a consequence of the Ferguson reflex, pain and stress levels are reduced.

When the outgoing or motor (efferent) sympathetic nerves are activated, strong uterine contractions, that can also elicit pain, are induced. In addition, the sympathetic nerves reduce the blood flow of the uterus.

The ingoing, afferent, sensory fibres of the sympathetic nerves are activated by strong uterine contractions which may elicit pain. Such stimulation can also increase the release of stress hormones (CRF and cortisol) and may in turn trigger the activity in the outgoing sympathetic nerves (the flight-or-flight stress system). Note that these stress effects are not necessarily a consequence of pain but are directly induced by nerve connections (axon collaterals) in the hypothalamus and brain stem. The sympathetic nerves that innervate the uterus may also be activated and cause prolonged uterine contractions and decreased uterine blood flow [18].

#### 1.2.1 Balance between the stress and the oxytocin system during labor (data cited and main references from Uvnäs Moberg et al., 2019 [18])

The oxytocin system and the stress system act independently during labor but may also inhibit the activity of each other. Oxytocin released within the brain during labor in response to activation of the Ferguson reflex modifies the stress reactions induced by labor by decreasing the levels of corticotropin releasing factor (CRF) in the PVN and of the sympathetic nervous system (the stress system). In addition, oxytocin also decreases pain and fear. However, if the activity in the stress system becomes too high, the opposite effect is induced and the activity in the oxytocin system and the parasympathetic nervous system are decreased. Such a shift in favour of the stress system may occur, for example, when the uterine contractions become too frequent, intense and painful. In this case the signalling of the ingoing sensory and sympathetic nerves from the uterus will give rise to such a strong activation of the stress system that the stress-buffering capacity of the oxytocin system is no longer sufficient. The activity of the oxytocin system may even be decreased by high activity in the stress system, since oxytocin release is decreased by stress.

#### 1.2.2 Strengthening of oxytocin release by support and tactile stimulation (data cited and main references from Uvnäs Moberg et al., 2019 [18] and Uvnäs Moberg et al., 2014 [23])

Oxytocin release during labor can be reinforced by physiological techniques in several ways e.g. by gentle activation of sensory nerves in the skin which stimulates oxytocin release and decreases stress levels and pain. Similar effects can be obtained by calming and supportive interactions. These types of interactions could be induced by the woman´s partner, birth companion or a midwife. Support and tactile stimulation (touch) can further activate the oxytocin system and thereby decrease levels of fear, stress and pain [18,23].

### 2.1 The psychological experiences of physiological childbirth (data cited and main references from Olza et al., 2018 [19])

A meta-synthesis of studies has been performed and published exploring women´s perceived experiences of physiological childbirth [19]. The aim was to search, retrieve and synthesise qualitative studies that included narrative data of women’s experiences of physiological childbirth. Physiological childbirth was defined as an uninterrupted process with no medical interventions in a supportive woman-centred environment. Data from the original studies which were analysed included quotes, interpretations and explanations [24].

Giving birth physiologically was described by women as an intense and transformative psychological experience that generates a sense of empowerment. Below follows a more detailed description of women´s psychological experiences during the different phases of physiological birth and a plausible explanation from a neuroendocrinological perspective [19].

### 2.2 Early labor: Social interaction, caring and nestbuilding (data cited and main references from Olza et al., 2018 [19])

We found in our previous paper that when women feel that labor has started, they inform other women from their social network. Some women feel excited and others describe experiencing a lovely feeling, even describing how things seem more beautiful than usual. At the onset of labor women expressed the need to continue with their usual routines. The examples they referred to included taking a shower, taking care of their children and their pets and being in the familiar environment of their home [19].

### 2.3 Advanced labor: Inner focus and need for support (data cited and main references from Olza et al., 2018 [19])

As the labor intensifies, women describe how they withdraw from the outer world and retreat into themselves. At this stage the focus of the birthing woman is on the physical task of the imminent birth of their baby and how to handle the increasing levels of pain. They might also want to move around and submerge themselves in warm water to help focus on the work of labor and relieve the pain.

As labor further progresses, and the intensity of contractions increases, women express their desire to be in a safe protective environment with supportive companions. They describe how important their partner can be to help them to cope as labor intensifies. At this time women often call for assistance and support to help them and make contact with their midwife and /or move to the hospital [19].

### 2.4 Labor as an altered state of consciousness (data cited and main references from Olza et al., 2018 [19])

As labor becomes even more intense women describe how they focus on the importance of living in that moment and time feels suspended. The perception of time and space changes, and women can experience intense feelings, which is compatible to an altered state of consciousness. Women may feel they are worlds apart from people in the same room, that the universe has narrowed to this one task they have to do. Some women mention transcendental experiences; of feeling part of the divine, the universe or gaining a deeper understanding of, or being a part of, nature.

### 2.5 Women become more active during pushing (data cited and main references from Olza et al., 2018 [19])

Towards the end of labor, the intensity of pain continues to increase and may override the pain relieving and calming effect of having a close person nearby. Some women feel they want to give up, they can’t do anything more. They feel that they cannot continue, expressing fears of death. Maximal levels of fear and pain are common just before pushing. Some women feel exhausted and deprived of energy.

With the urge to push, women often become alert and more active, as if they are “coming back to push”. Women re-enter the outer world environment and time no longer feels suspended [19].

### 2.6 Joy and pride-immediately after the baby is born (data cited and main references from Olza et al., 2018 [19])

Immediately after the baby is born, some women experience an urgent need to explore their baby in detail and to assure themselves that their baby looks normal. Women may describe feelings of ecstatic joy in reaching this glorious zenith and express feelings of spiritual closeness and gratitude. They describe how their ability to positively use their pain to achieve normal birth influences their confidence in becoming a mother. This unique and powerful experience was juxtaposed with the need for a sense of peace and routine to ground them in the new reality of motherhood. Women with other children often want to connect their newborn together with their siblings and other family members commencing bonding and attachment to the new family member. Women describe a sense of being enveloped with their new baby in a protective environment by their family where the new baby is showered with love including hugs and kisses [19].

### 2.7 Postpartum—A sense of transformation and empowerment (data cited and main references from Olza et al., 2018 [19])

Besides wondering at the uniqueness of the birth experience, expressing relief and joy at meeting their baby, and their feelings of childbirth as being their greatest, unparalleled achievement, women may describe a sense of transformation. Some women describe themselves as a changed person in the sense that they feel stronger, empowered, and ready to meet the demands of the newborn. Overall the journey through childbirth means a growth in personal strength, a transformation that leads to an empowered-self.

### 3 Birth as a psychological journey facilitated by neurohormonal mechanisms

In this section the information from the two previous papers is integrated in order to give a neuroendocrine explanation to the subjective experiences and the behaviors described by the birthing women (Uvnäs Moberg et al., 2019 [18] and Olza et al., 2018 [19]).

#### 3.1 Early labor: Social interaction, caring and nestbuilding, proposed mechanisms involved

The behaviors, feelings and perceptions during early labor are likely to be related to the increase of oxytocin levels that occurs in the circulation and the brain [18]. Oxytocin stimulates friendly social interaction [25], and therefore the need for women to share the onset of labor their family and close friends may be a consequence of increased oxytocin levels. Working with practical aspects of the household could also be expressions of oxytocin facilitated care-taking and nest-building behaviours.

The increased levels of oxytocin in the brain could also trigger the feelings of well-being, happiness and a positive mood described by some women.

#### 3.2 Advanced labor: Inner focus and need for support and nestbuilding, proposed mechanisms involved

During this phase of labour women often reach out for somebody to provide them with physical contact and mental reassurance. The birth companions may offer supportive physical contact and verbal reassurance in order to reinforce the woman´s trust in her own capacity to labor. These interactions also help reduce fear, stress and pain by increasing the activity in the oxytocin system. As presented above, the brain is continuously informed by two parallel systems or nervous circuits sending information from the uterus to the brain: (a) the “parasympathetic” oxytocin system (Ferguson reflex), giving rise to oxytocin release and increased parasympathetic activity and (b) the “sympathetic” pain fibres, giving rise to pain and increased activity in the stress system. As labor contractions become stronger, the laboring woman may need a person to help them regulate increasing levels of pain, stress and fear, by being close to them. Physical contact and mental support activate the oxytocin system and therefore reduces the pain and lowers the activity in the stress system. Women intuitively ask for physical closeness, contact and reassurance, to help maintain the balance between the oxytocin and the stress systems. Activation of sensory nerves from the skin play an important role in the release of oxytocin in response to closeness [23].

This universal need for a caring approach includes social and professional support: provided by the birth companion or partner, and the woman’s midwives (or, in some cases, doctors). Support from midwives helps women manage the vulnerability they experience during labor as well as the experience of fear and pain. Therefore, when women undergo physiological birth, the most natural pain and stress relief is their own oxytocin release, which can be potentiated by touch and reassurance, from the maternity care provider, birth companion or partner [23,26].

Oxytocinergic nerves, increasingly activated during labor, connect to brain areas involved in pain control, such as the periaqueductal grey (PAG) and the spinal cord and areas involved in reward and wellbeing [27]. Oxytocin released within the brain inhibits pain via opioidergic mechanisms involving activation of Mu opioid receptors [23]. Oxytocin-induced pain relief may be linked to the amnesic effect, which helps the new mother forget the intensity of labor on the days that follow.

#### 3.3 Labor as an altered state of consciousness, proposed mechanisms involved

Several signalling systems may be involved in the altered state of consciousness. The opioid system is likely to be involved, activated by oxytocin to give endogenous pain relief and wellbeing. Serotonergic, catecholaminergic and dopaminergic mechanisms may also be involved [27].

#### 3.4 Women become more active during pushing, proposed mechanisms involved

The increased activity and return to the outer world could be caused by the catecholamine surge that occurs at this time during birth [28,29]. The catecholamine surge is triggered by a very high intensity of the signalling of the uterine sensory nerves mediating pain and stress. Consequently, a very strong activation of the noradrenergic neurons in the locus coeruleus occurs, which results in high levels of noradrenaline and adrenaline, which in turn increases alertness and activity in the HPA axis and of the sympathetic nervous system.

#### 3.5 Joy and pride-immediately after the baby is born, proposed mechanisms involved

The immediate instinctual checking of the baby is likely to be facilitated by the catecholamines surge in the maternal brain that is induced during birth. The postpartum euphoria may be linked to oxytocin-mediated dopamine release in the reward centre of the brain [30]. As shown in our paper 1 [18], in which data on oxytocin levels during labor were reviewed, oxytocin levels exhibit a 3-4-fold rise in the circulation as the baby is born and most likely a parallel rise of oxytocin occurs in the brain. This rise of oxytocin levels may be linked to an increased release of dopamine, among other effects [18]. Oxytocin released during birth, and during skin-to-skin contact after birth, promotes interaction and attachment between mother and baby in many ways [31]. Women´s need to reunite the family may be an expression of the prosocial and pro-attachment effects of oxytocin. The need for seclusion and peaceful contemplation may be linked to the powerful anti-stress effects induced by oxytocin released in response to touch and warmth after birth and during skin to skin contact between mother and baby [23].

#### 3.6 Postpartum—A sense of transformation and empowerment, proposed mechanisms involved

The euphoria and the sense of transformation coincide with and are likely related to the very high levels of catecholamine and of oxytocin and dopamine in the brain immediately after the birth. Such high levels may not be achieved in any other circumstance during life [32,33], meaning that, the function of the brain is out of the ordinary and exceptional during birth. These high levels of oxytocin may, in addition to promoting the release of dopamine, also cause activation of serotonin pathways and vice versa.

Women subjectively report changes in how they feel and regard themselves as their situation has changed. After giving birth, women report lower levels of anxiety and higher levels of social interactive behaviour, according to research using the Karolinska Scales of Personality [34]. These personality changes facilitate attachment and mothering and are induced by oxytocin. Women link their pride in coping with labor to feeling strong and confident, and experiencing a positive start to a new motherhood.

## Discussion

### The integrative neuro-psycho-social model of childbirth

**Fig 1:**
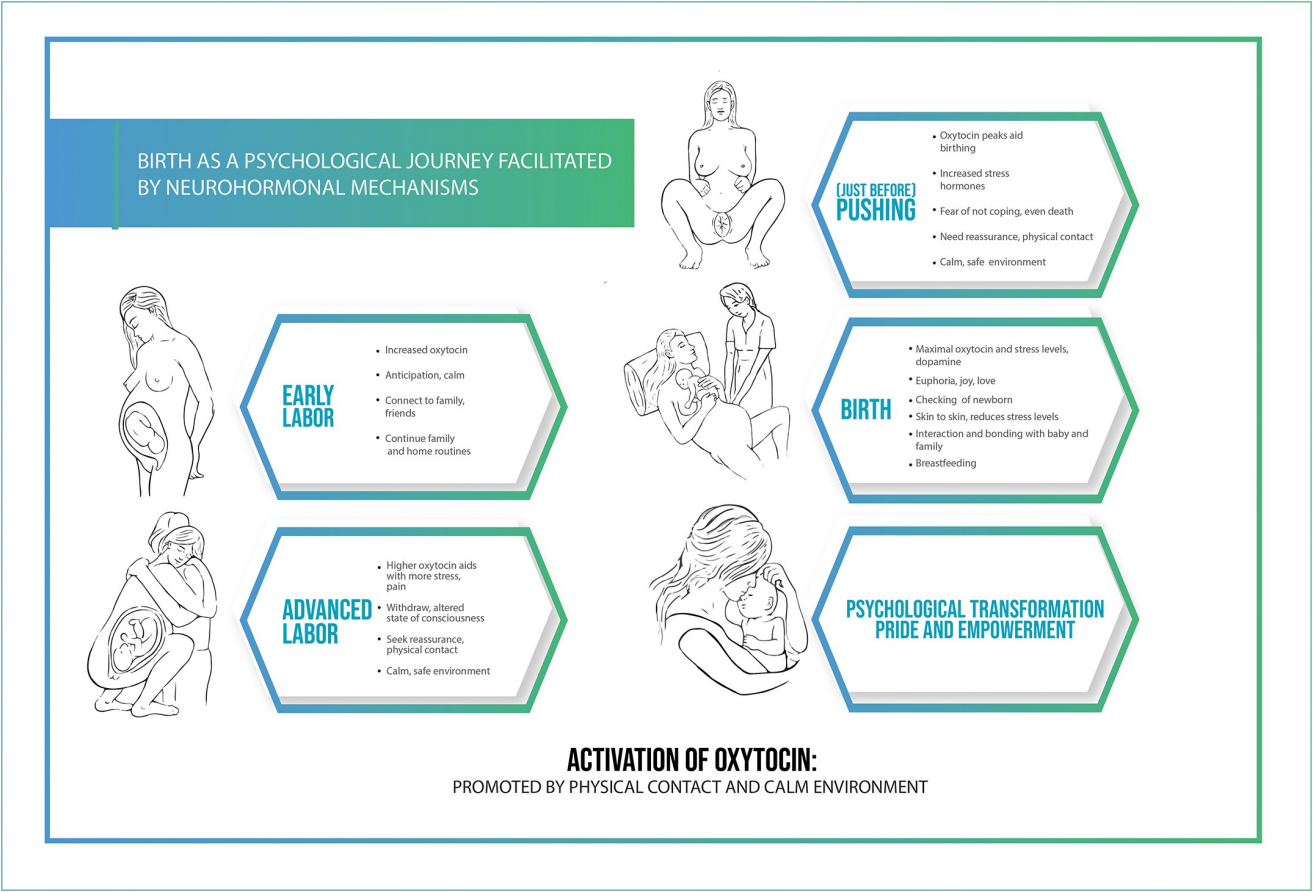


Our paper offers a new model of childbirth that integrates neuroendocrinological, physiological and psychosocial aspects using the lens of women’s subjective experiences following a physiological birth. In summary oxytocin does not only stimulate uterine contraction during labor, it also influences the mothers´ experiences, behavior and physiology in order to facilitate birth. The role of oxytocin in the different stages of birth is illustrated in Fig 1.

By integrating the neuroendocrine events during childbirth with psychological experiences a wider picture of human childbirth unfolds. In this model, the neurobiological processes correlate with, and facilitate the intense and transformative psychological experience during transition to new motherhood. The importance of social and professional support during childbirth underlines the need for human touch and reassurance that is provided by both companions and maternity care providers. Oxytocin linked anti-stress effects which antagonizes pain and the fight-flight response are of great importance in these situations [35].

This model integrates the effects of the complex interrelations between physiological, psychological, and social factors and it highlights the crucial role of one-to-one support, a hallmark of midwifery care [36]. The physiological necessity for social support to reduce labor stress and pain leads to a reconsideration of many aspects of modern maternity care.

The integrative perspective presented in this paper highlights the importance of both close attention to the laboring woman’s emotional state and an awareness of the neuroendocrinology involved in labor and birth. It suggests rethinking how women are cared for and supported, from pregnancy through to the postpartum period. These new perspectives have many important implications for maternity care professionals, mental health specialists, researchers and for women and society.

In order to implement these wider perspectives into maternity care, it is essential that maternity care providers are educated and enabled to support these processes. Specifically, paying attention to how the laboring woman feels, acts, talks and behaves, is a key clinical aspect of labor progress. A key message for maternity care providers is to: “protect, observe and listen to birthing women and help them listen to their bodies”. This highlights the importance of recognizing and responding to the laboring woman’s emotions and cognitions, which will empower her and increase her sense of well-being while managing the own emotions and being aware of the uniqueness of each experience [37,38]. Training and supporting maternity care providers to meet each woman´s unique needs during childbirth can increase her satisfaction with caregiver support, her relationship to, and feelings for, her baby, as well as promoting breastfeeding initiation and duration thus facilitating adaptation to motherhood [39].

It is important for maternity care providers to know that the central release of oxytocin, and its positive effect, can be modified by environmental factors. Stress and frightening situations and surroundings increase the activity in the stress system (HPA axis and the sympathetic nervous system) and decreases the activity of parasympathetic nervous activity and the decrease oxytocin release [23].

There may be significant individual differences in relation to what is experienced as stressful for an individual woman in labor. The evidence suggests that some women find the following experiences stressful: the presence of an unfamiliar maternity provider, restriction to bed, vaginal examinations, being exposed to strangers or listening to insensitive and rude comments, episiotomy, forceps or vacuum birth [40,41].

Stress during labor and birth, may inhibit oxytocin release, causing increased pain and fear, which in turn may negatively affect labor progress, leading to a cascade of interventions. Medical interventions may also interfere with oxytocin release. For example, epidural anaesthesia blocks oxytocin release, including in the brain, which reduces the activity of some of the adaptive neuroendocrine mechanisms [42]. Some studies have shown that women with epidural anaesthesia may not experience the usual beneficial personality changes following physiological birth, such as reduced anxiety and tension and increased sociability [43]. Further, despite women having freedom from pain with epidural anaesthesia, they may in spite of pain relief not report increased wellbeing in comparison to women without epidural anaesthesia. Women with pain relief may also respond to their newborns differently [43].

These data further support the need for maternity care that optimizes the function of the neuroendocrine processes for the laboring woman and her baby, even when birth interventions are needed [44,45]. Individualized emotional support empowers the woman, and increases her chance of a positive birth experience, even if the birth is protracted or requires maternity-care interventions [46]. If the woman’s partner is able to offer support, then it is essential that s/he is supported and enabled to be physically present in the room throughout labor and birth [47,48].

The environment for labor and birth must also be considered in order to reduce stress. Labor wards and obstetric theatres that look pleasant and home like will be more welcoming and comfortable [49], not only for the woman and her companion, also for her maternity care providers [50,51].

### Implications of this model of childbirth

#### The unique state of consciousness during childbirth

During physiological childbirth, women describe a transient alteration of consciousness and they perceive time and space differently [52]. Women may have near-death experiences or experience transcendental stages. Some feel their sense of self dissolve, others feel somehow connected to other women laboring at the same time and some feel connected to a higher entity and indicate that they have gained a deeper understanding of life [53,54]. After physiological birth women can feel transformed and empowered, and these changes are enduring. This empowerment can help women feel more capable to nurture and protect their newborn. The spiritual growth described probably leads to other positive changes that will require further research [55].

This description of women´s experiences during labor and birth and its potential for transformation resembles descriptions of mystical states of consciousness. Classically these states have been achieved through meditation and religious practices (including dancing, praying and fasting) or through intake of substances with hallucinogenic properties such as psilocybin or LSD, which interact with serotonin receptors [56]. Childbirth has not been mentioned in those classical descriptions. The experience of spontaneous altered states of consciousness may well be a hallmark of physiological childbirth in humans and therefore its research may offer a unique opportunity to understand consciousness and transcendental growth. Researchers have previously reported spiritual growth as common during childbirth indifferent cultures [53,57]. This knowledge is important to include in birth preparation courses and consultations.

#### Implications for understanding of traumatic birth

Understanding the positive, transformative effects of physiological childbirth also increases our understanding of the long-lasting symptoms and distress many women suffer after traumatic childbirth [58]. Inadequate support or mistreatment from health professionals can lead to a negative birth experience where women feel abandoned, immobilized, and dismissed [59]. The neurohormonal environment in the maternal brain during birth may increase the chance of both strong positive and negative experiences. Negative experiences linked to strong stress reactions may become turned in to long lasting or “imprinted” effects, which could contribute to the high rates of PTSD symptoms following childbirth [6]. Some women describe grief and sorrow after planned cesarean section or other maternity care interventions which may be related to the loss of the transformative and empowering experience that comes with physiological childbirth under optimal care. Women´s negative experiences may impact the functioning of the woman and the whole family [60,61]. Listening to and validating women´s experiences and feelings after a traumatic childbirth may help prevent the occurrence of PTSD.

#### Social implications of the integrative neuro-psycho-social model of childbirth

The view of labor and birth presented in this paper challenges the prevailing social and medical view of childbirth as an experience of avoidable pain that is better faced with epidural analgesia or even managed by cesarean section. It is urgent that women are supported and empowered to strengthen their own capabilities by midwives [62], and given a rightful place as decision-makers in pregnancy, labor and birth. If childbirth has evolved, as suggested, to give women a powerful psychological reward that includes transformation, empowerment and pleasure, it is important that women are provided with this knowledge. What we tell young people, women and men, about birth is a social and feminist issue [63,64]. Yet we should be cautious to avoid unrealistic and idealistic expectations, which may increase pressure on women to achieve physiological birth. Rather, we must empower women with knowledge and understanding of the possibilities for transformation and reinforce the importance of good support and midwifery care.

### Suggestions for further research

We suggest this research could be continued by inviting women from diverse cultural backgrounds to contribute to the discourse (as existing psychological studies on physiological birth are scarce with very selected samples). An analysis of the experiences of partners and other support people is also valued to widen this perspective and allow for inclusion of social and cultural diversity into the model.

Future research could focus on the piloting of our new model and exploring differences between primipara and multiparous women, as well as with women with a previous history of trauma or mental illness. Neuroimaging studies could also contribute to this model increasing our understanding of intrapartum brain process and or changes. Further, the contextualization in different cultures and medical systems could provide additional, relevant information to the transitioning processes towards a more humanistic and respectful model of maternity care. In such studies, women and midwives should be empowered and enabled to actively participate in shaping research projects and to voice their experiences, both positive and negative, in the public sphere.

## Conclusions

Neurobiological processes, orchestrated by endogenous oxytocin release, facilitate labor and birth. These processes are also associated with the intense and transformative psychological experiences of labor, which facilitate transition to motherhood. The fact that human touch, support and reassurance facilitate oxytocin mediated reduction of fear, stress and pain as well as oxytocin mediated promotion of joy and empowerment explain why one-to-one support during childbirth, a hallmark of midwifery care, is crucial.

The physiological importance of social support for reduction of pain and stress during labor prompts a reconsideration of many aspects of modern maternity care. There is sufficient evidence to increase advocacy for improved maternity care and for promotion of midwifery one-to-one support in labour. This information also calls for a more feminist and humanistic attitude regarding labour and birth from public institutions and health professionals worldwide.
